# Gender differences in medical students’ motives and career choice

**DOI:** 10.1186/1472-6920-12-82

**Published:** 2012-08-23

**Authors:** Phil JM Heiligers

**Affiliations:** 1 NIVEL - Netherlands institute for health services research, PO Box 1568 3500 BN, Utrecht, The Netherlands; 2 Utrecht University, Faculty of Social sciences, PO Box 80.140 3508 TC, Utrecht, the Netherlands

## Abstract

**Background:**

The main subject is the influence of gender and the stage of life on the choice of specialty in medical education. In particular we looked at the influence of intrinsic and external motives on this relationship. The choice of specialty was divided into two moments: the choice between medical specialties and general practice; and the preference within medical specialties. In earlier studies the topic of motivation was explored, mostly related to gender. In this study stage of life in terms of living with a partner -or not- and stage of education was added.

**Methods:**

A questionnaire concerning career preferences was used. The online questionnaire was sent to all student members of the KNMG (Royal Dutch Medical Association). 58% of these students responded (N = 2397). Only 1478 responses could be used for analyses (36%). For stipulating the motives that played a role, principal components factor analysis has been carried out. For testing the mediation effect a set of regression analyses was performed: logistic regressions and multiple regressions.

**Results:**

Although basic findings about gender differences in motivations for preferred careers are consistent with earlier research, we found that whether or not living with a partner is determinant for differences in profession-related motives and external motives (lifestyle and social situation). Furthermore living with a partner is not a specific female argument anymore, since no interactions are found between gender and living with a partner. Another issue is that motives are mediating the relationship between, living with a partner, and the choice of GP or medical specialty. For more clarity in the mediating effect of motives a longitudinal study is needed to find out about motives and changing circumstances.

**Conclusions:**

The present study provides a contribution to the knowledge of career aspirations of medical students, especially the impact of motivation. Gender and living with a partner influence both choices, but they are not interacting, so living with a partner is similarly important for male and female students in choosing their preferences. Moreover, external and intrinsic motives mediate this relationship to a greater of lesser degree. First stage students are influenced by life-style and intrinsic motives in their choice of general practice. For second stage students, the results show influences of life-style motives next to profession-related motives on both moments of choice.

## Background

Insight into the career choice of medical students has become a real issue as the student population is changing towards a majority of female students. The consequences of this increasingly female student population are not only being studied in the Netherlands, where this study was performed [1-3] but also in other countries [4-7]. The focus of the current study is to provide more insight into the impact of gender and, the stage in the student’s life, on his or her preferences for a medical specialty. In addition, this study examines the influence of the gender and the stage of life on the motives that direct a student’s career choices. We investigate the effect of external and intrinsic motives. These motives are expected to mediate the relationship between gender and the stage of life on the one hand, and future specialty on the other.

Earlier studies on this topic have shown that the choice of specialty is related to gender. Male students opt for technical and instrumental oriented specialties and female students are more relationship oriented [8]. For example, male students more often choose surgery as a specialism, whereas female students tend to prefer to be a general practitioner. Again, this phenomenon was found both in the Netherlands [3,8-10] as well as in other countries [6,11-14].

Next to gender, the influence of students’ stage of life is an important issue. Most students in our study are young, and may therefore be less likely to have a permanent partner. However, we expect that having a partner will have some impact on the choices for the future.

To some extent the ideas of the partner and the perspectives of balancing work and family will influence choices in specialties and might also influence the importance of several motivating aspects for the choice of specialty [1].

The main question in this study is to what extent are gender and the stage of life related to the choice of specialty and how intrinsic and external motives influence this relationship.

### Theoretical background

In this study we focus on several perspectives that in earlier studies have been indicated as important contributions to explain a career choice. Our effort is to explain the relationship between these perspectives: the gender perspective, the perspective of the stage of life and the motivational perspective, in the decision of students to choose a particular career.

#### Gender perspective

Gender refers to aspects of sex related to behaviour and identity, which go beyond biological differences [15]. Although the participation of women in the labour market has increased, segregation can still be found between male and female professions or specialties. This segregation is mostly in favour of the professions or specialties which are typically male [16,17]. Expectations based on these patterns influence individual career choices of men and women, which is also the case for individual medical students and their expectations [3,9,18,19]. In several studies it was found that men, more often than women, choose the surgical specialties [3,6,9-14]. Van der Velden et. al. [20] found that in spite of the fact that women cover more than half of the Dutch medical student population; only 13% of all working surgery specialists are female. In other specialties 30% of all specialists are female, on average, with exceptions for gynaecology (40%) and paediatrics (53%), which are traditionally associated with the reproductive gender role of woman [10,21].

From the gender perspective, we expect that male medical students are, more than female students, focused on specialties with technical and instrumental characteristics. Female students, more than men, will focus on specialties with opportunities for relational aspects.

#### The stage of life perspective

Next to gender, the stage of life is related to the choice of specialty [22]. Changes and transitions between the stages of life, such as marriage (or living with a partner), or becoming a parent, have an impact on social roles, relationships and choices for the future [23]. Expressed in years, medical students have a rather long period of study and preparation before they start a career as medical specialist. In the last years of their life as a student, some transitions in their stage of life might take place, such as having a partner or even a child. These experiences can influence the choice of a specialism, because ideas of a partner or responsibilities towards family life, can direct the specialty choice.

Furthermore, these students are confronted with conflicting roles. Being a student and partner can limit their preferences towards less challenging specialties [24]. We expect medical students living together with a partner will make other, family-friendly, specialty choices.

Also, we expect junior students to choose more idealistic options, perhaps the most appealing specialties, as opposed to older students who may choose more realistic options that is specialties matching their other roles such as partners or parents.

#### Motivational perspective

Men and women do not only differ in their preference for a specialty, but also in the motives for their choice [3,9,13,18,25,26]. Generally, male students are more motivated by salary, status and the opportunity to implement technical activities. Female candidates are motivated by humanist and altruistic reasons [19,25,27]. Theoretically, intrinsic motivation is based more on personal involvement with the content of the work and external is based more on involvement with techniques and innovation [28]. Intrinsic motivation is related more to specialties in which patient-related activities make up a major component. This is mostly preferred by women. External motivation is related to specialties characterized by technical skills and physical exertion, which are more attractive for male students [9]. We expect male students to be more externally motivated, focused on salary and status and, within the specialty, on applying technical skills. Whereas the motives of female students will be more intrinsically focused on specialties with options for intensive patient contacts.

Specific characteristics of several specialty choices seem to match with different types of motives. Intrinsic motives are related to personal and continuous care, such as relationships in GP practices. Specialties related to surgery seem to be more related to external motives, such as status and income and a high workload [1,4,29]. These relationships imply that motives could have a mediating role between gender and specialty choice.

It seems that the stage of life, and specific experiences, such as becoming involved in a permanent relationship, has an impact on motives, which consequently influences specialty choice [4]. Therefore, it can be expected that the stage of life is not only directly related to specialty choice, but mediated by motives.

#### Summarizing: the conceptual model

The conceptual model will give an insight into the choice of specialty of medical students which assumes several influences related to gender and the stage of life. In the model specialty choice will be divided into: (1) the choice of medical specialties or general practice and (2) the preference within medical specialties, divided into surgery or non-surgery specialties.

**Figure 1:**
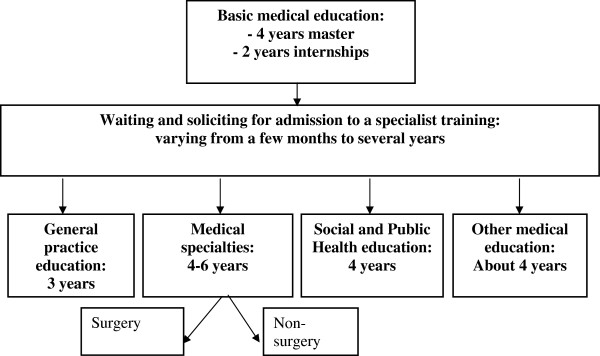
Diagram of medical education in the Netherlands.

After medical students have finished their basic medical training most of them (85%-90%) continue their education with a specialist training [8]. In Figure1 the time plan of several medical studies is given, which also gives an insight into at what point choices are made. 

The first choice in further training is between training for general practice or for a medical specialty. On average, male students choose, more often than women (63% against 57%), a medical specialty [8].

A second choice is focused on all medical specialty training programmes. In the Netherlands, women are under-represented in surgery-related specialties, 10% of women against 90% men [20,30]. A GP training is generally chosen by 20% of the medical student population and women tend to choose the option more often. But, since the female population among medical students is growing, the number of women with a preference for a medical specialty is also increasing [30]. Despite recent changes in the overall population of medical students we still expect that male students will choose, more often, to train for medical specialties, and especially surgery-related specialties. GP training will be chosen by women more than by men.

In the conceptual model it is expected that a direct relationship exists between gender and the stage of life on the one hand, and the choice of specialty on the other hand. Beside this direct relationship, gender and stage of life are expected to relate to the motives of students in making their choices. The motivation of students is subdivided into: (1) Extrinsic motivation which is related to their profession and made up of aspects such as technical skills, status, and income, and; (2) Extrinsic motivation which is related to life-style including aspects such as working hours, controllable life-style, and having children, and finally; (3) Intrinsic motivation, which is related to personal involvement and patient-related activities. A partial mediating influence of motives is expected on the relationship between gender and the stage of life on the one hand and specialty choice on the other hand.

## Methods

### Participants

A questionnaire concerning the career preferences of medical students was used. The questionnaire was sent by e-mail to all student members of the KNMG (Royal Dutch Medical Association).

The response was 58% (N = 2397). Only 1478 students had filled in the questionnaire sufficiently for the analyses, which is 36% of the population. This response group can be considered representative for the research population, since the comparison of demographic characteristics showed only a slight over-representation of female students (72% against 68%).

### Variables

#### Independent variables

*Gender* was measured by the variable sex: 0 = male, 1 = female. S*tage of life* has been constructed by measuring two variables. In the first place the variable *living together* is divided into two levels: 0 = `not living together’, 1 = `living together’. Living together was measured by being married or living with a partner. The second variable constructing the stage of life is *educational stage.* Two stages of basic medical training are constructed. In the first stage students are included who have studied for three years and gained between 0 and 180 credits – the maximum in the Dutch system over three years. In the second stage students are included who studied more than three years and had more than 180 credits. The first stage is scored as 0 and the second stage = 1.

#### Dependent variables

*Choice of continuing medical education was divided into two* preferences: medical specialty and general practice and the category: ‘do not know’. For the analysis the variable of *continuation choice is* binary measured with 0 = medical specialty and 1 = general practice.

##### *Medical specialism preference*

Students could indicate their preference for a type of medical specialty within five main categories, which are very common in the Dutch medical profession system: supportive, surgery and internal medicine as hospital oriented and psychiatry and ophthalmology as both hospital and/or solo practice and a sixth category of ‘others’ was added. For all hospital oriented categories and the ‘other’-category students could further fill in their self-defined very specific specialist preference. These string variables resulted in a very long list of possible medical specialties. In order to have substantial numbers of students on different preferences we divided into specialties for which surgery skills are required and those requiring no surgery skills. The variable m*edical specialty preference* has two categories 0 = non-surgery specialty, 1 = surgery specialty.

#### Mediating variables

##### *Motivation*

Respondents could choose among 15 motives which could support specialty choice. On a five-point scale, the importance of each motive could be indicated. To retrieve clusters of motives, factor analysis was applied (Table1). It provided three factors on: 1. Life-style characteristics (with the items `domestic situation’, `control of life-style’, ’having small children’, `flexible working hours’); 2. Profession-related characteristics (with the items `high status’, `favourable circumstances at work’, `high income’ and `career and promotion expectancies’); and 3. Personal involvement (with the items `interest in the field’, `patient oriented and meaningful’, `internal motivation’ such as ‘achieving happiness’ and the `contents of the future profession’). Three items were not loading on one of the factors: ’the opportunity of rotation in the field of subspecialty’, ‘learning special skills’ and ‘availability of training’. These items are not used in analyses. In addendum 1 the motivation items are presented.

**Table 1:** Results of the Principal components factor analysis (Varimax rotation with Kaiser Standardization has been used)

**Factor**	**Items**	**Factor loadings**		
		**1**	**2**	**3**
Life-style characteristics (external)	Domestic situation	0.89		
	Having small children	0.88		
	Control on life-style	0.82		
	Flexible working hours	0.81		
Profession-related characteristics	Status		0.87	
(external)	Favourable circumstances at work		0.82	
	Income		0.77	
	Careers and promotion expectancies		0.73	
Personal involvement	Interest in the field			0.80
(intrinsic)	Patient-oriented and meaningful			0.78
	Internal motivation such as achieving happiness			0.70
	Contents of the future profession			0.63
R ²	0.64			

### Analyses

#### Mediation analyses

It is expected that the relationship between gender and the stage of life on the one hand, and the choice of continuation and specialty choice on the other hand, is mediated by the motives supporting the choice. The following set of regression analyses are performed separately for both the independent variables: continuation choice and medical specialty choice [31]. These are: (1) a binary logistic regression of gender and the stage of life conditions (living together and stage of education) on the choice; (2) with multiple regression the relationship between gender and the stage of life conditions on the one hand and the mediator variables (motives) on the other hand are examined; (3) a binary logistic regression on the relationship between mediator and the choice is performed; and (4) a binary logistic regression of gender, stage of life conditions and the mediator on the preferred choice. Logistic regressions are performed in four steps to meet the expectations we hypothesized in the theoretical background.

All mediating steps are separately analysed for students in the first and the second stage of education because we expect some cohort effects related to the stronger influence of the *stage of life* in the second stage. In all analyses, we controlled whether gender was a moderator for living together and for the stage of education, but these interactions were not found.

#### Factor analysis

For stipulating the motives that played a role in the choice of a specialty, principal components factor analyses have been carried out on the question concerning the importance of mentioned motives for the choice of a specialty. Varimax Rotatione with Kaiser normalization is used [32]. Only the items with a factor contribution of 0.40 or higher on 1 factor, but which have contributed low on the remaining factors, are included.

Three factors were traced and they explain together 64% of the total variance. Factor 1 concerns external motivation on life-style characteristics with four items mentioned in Table1.

(Cronbach’s Alpha = .89). Also factor 2 is external in nature, focused on professional characteristics (4 items, Cronbach’s Alpha = .82). The third factor constitutes personal involvement (4 items, Cronbach’s Alpha = .74).

## Results

The correlations between gender, the stage of education, living together, the motives and specialty choice are given in Table2. These results are in line with outcomes of earlier studies [3,8,9,19,25,27,33]. 

**Table 2:** Means, standard deviations and Pearson correlations of all variables

		**M**	**SD**	**1**	**2**	**3**	**4**	**5**	**6**	**7**
1	Gender									
2	Stage of education			.03						
3	Living together			.05	.20**					
4	Choice of continuing medical education			.10**	.01	.12**				
5	Medical Specialty preference			-.10**	-.08	-.14**	.00			
6	Life-style characteristics- external motivation	3.61	.86	.18**	.06	.13**	.26**	-.11**		
7	Professional characteristics - external motivation	3.35	.77	-.19**	-.06*	-.02	-.19**	.18**	.15**	
8	Personal involvement – intrinsic motivation	4.33	.60	.08**	.15**	.07**	.09**	-.06	.22**	.11**

* *p* < 0.05.; ** *p* < 0.01.

The characteristics of the population are given in Table3. As expected, students in the second stage of education are older and are more often living with a partner. From a considerable number of students their stage was unknown. On average their age is comparable to the age of students in the second stage.

**Table 3:** Characteristics of all respondents (N = 1478), respondents of the first stage of education (N = 679) and the second stage of education(N = 396)

**Characteristics**	**All respondents**	**Respondents first education stage**	**Respondents second education stage**	**Respondents unknown education stage**
Average age	22.8	20.6	23.9	23.8
Gender: men	27.8%	28.3% (N = 192)	26.0% (N = 103)	28.8% (N = 116)
women	72.1%	71.7% (N = 487)	74.0% (N = 293)	71.0% (N = 286)
Living together: men	16.5% (N = 67)	8.9% (N = 17)	23.3% (N = 24)	6.5% (N = 26)
women	20.6% (N = 220)	12.3% (N = 60)	28.3% (N = 83)	19.1% (N = 77)
Stage of education: first stage	45.9% (N = 679)			
second stage	26.8% (N = 396)			
unknown	27.3% (N = 403)			
***Choice of continuing medical education:***				
General practice	9.7% (N = 144)	8.2% (N = 56)	10.1% (N = 40)	11.9% (N = 48)
Medical specialty	61.9% (N = 915)	57.4% (N = 390)	67.2% (N = 266)	64.3% (N = 259)
do not know	21.6 (N = 319)	29.2% (N = 198)	14,1% (N = 56)	16.1% (N = 65)
other	5.7% (N = 84)	3.5% (N = 24)	7,9% (N = 31)	5,2% (N = 21)
missing	1.0% (N = 16)	0.4% (N = 3)	0.8% (N = 3)	2,5% (N = 10)
***Medical specialty choice:***				
Surgery specialty	21.5% (N = 318)	20.2% (N = 137)	23.7% (N = 94)	21.6% (N = 87)
Non-surgery specialty	25.2% (N = 373)	19.4% (N = 132)	31.1% (N = 123)	29.3% (N = 118)
others	17%% (N = 148)	18.7% (N = 73)	13.9% (N = 37)	14.7% (N = 38)
missing	3.0%) (N = 32)	5.4% (N = 21)	1.6% (N = 4)	2.7% (N = 7)

The percentage of men and women living together is not very different in each stage of education, but in the population of which their stage is unknown relatively more women than men are living together.

Relatively more students in the second and in the unknown stage made a choice for continuing medical education as well as for a medical specialty compared to students in the first stage. Almost 30% of first stage students does not know what choice they will make and for second stage students this is 14%. From those who chose medical specialty almost 19% of the first stage students and 14% of the second stage students could not make a choice yet between surgery or non-surgery.

### Gender, choice and motivation

The choice of continuing medical education between medical specialties or general practice is, as expected, influenced by gender (see Model 1, Table4). Compared to women, men are less likely to prefer general practice to medical specialty. However in separate analyses for the stage of education this ‘gendered’ impact was not found.

**Table 4:** **Logistic Regression on Choice of continuing medical education**^**1 **^**for all students, first stage students and second stage students (B-values, Odds Ratio, Wald statistics)**

	**All(N = 699)**						**First stage (N = 408)**						**Second stage (N = 291)**					
	**Model 1**			**Model 2**			**Model 1**			**Model 2**			**Model 1**			**Model 2**		
	***B***	***Exp (B)***	***Wald***	***B***	***Exp (B)***	***Wald***	***B***	***Exp (B)***	***Wald***	***B***	***Exp (B)***	***Wald***	***B***	***Exp (B)***	***Wald***	***B***	***Exp (B)***	***Wald***
Gender ^2,5^	0.55	1.74	4.09* ^**a**^	−0.02	0.98	0.00	0.46	1.58	1.70	0.00	1.00	0.00	0.72	2.04	2.63	0.04	1.04	0.007
Stage of education ^3^	−0.02	0.99	0.00	−0.14	0.87	0.31												
Living together ^4,5^	0.67	1.95	6.69*	0.27	1.30	0.84	0.74	2.09	4.01*	0.64	1.90	2.63	0.71	2.04	4.00*	0.35	1.42	0.83
Profession-related motives (external)				−1.06	0.35	38.12***				−0.21	0.81	1.33				−0.70	0.50	9.18**
Life-style motives (external)				1.18	3.24	41.52***				0.91	2.48	15.37**				0.97	2.63	13.39***
Personal involvement (intrinsic)				0.64	1.90	6.81**				0.79	2.20	5.12*				0.64	1.90	3.16
Nagelkerke Pseudo R²	0.03			0.23			0.03			0.15			0.043			0.19		
Chi² (df = 3 & df = 6)/ ^b^ Chi² (df = 2 & df = 5)	11.37**			93.40***			^b ^5.67			^b^33.75** *			^b ^7.02*			^b^32.02***		

* *p* < 0.05; ** *p* < 0.01; *** *p* < 0.001.

^a^ p = 0.046.

^1^ medical specialty = 0; general practice = 1; ^2^ 0 = male; 1 = female; ^3^ first stage = 0; second stage = 1; ^4^ 0 = not living together; 1 = living together.

^5^ Controlled for gender x living together*: p > .05*.

In their preference for a medical specialty men choose less often a non-surgery specialty as opposed to a surgery specialty compared to women (Model 3 in Table5). For students in the first stage this preference in medical specialty was not found.

**Table 5:** **Logistic regression on Preference for Medical specialty**^**1 **^**for all students, first stage students and second stage students (B-values, Odds Ratio, Wald statistics)**

	**All (N = 462)**						**First stage (N = 253)**						**Second stage (N = 209)**					
	**Model 3**			**Model 4**			**Model 3**			**Model 4**			**Model 3**			**Model 4**		
	***B***	***Exp (B)***	***Wald***	***B***	***Exp (B)***	***Wald***	***B***	***Exp (B)***	***Wald***	***B***	***Exp (B)***	***Wald***	***B***	***Exp (B)***	***Wald***	***B***	***Exp (B)***	***Wald***
Gender ^2,5^	−0.57	0.57	7.98**	−0.32	0.72	2.29	−0.52	0.60	3.77	−0.33	0.72	1.42	−0.60	2.04	3.87*	−0.14	0.87	0.18
Education stage ^3^	−0.28	0.76	2.08	−0.18	0.83	0.85												
Living together ^4,5^	−0.68	0.51	6.40*	−0.59	0.55	4.65*	−0.72	0.49	3.25	−0.70	0.50	3.00	−0.52	2.04	2.11	−0.27	0.76	0.49
Profession related motives (external)				0.48	1.62	12.09***				0.37	1.45	3.78				1.09	2.98	16.69***
Life-style motives (external)				−0.26	0.77	4.50*				−0.20	0.82	1.48				−0.57	0.57	6.80**
Personal involvement (intrinsic)				−0.24	0.79	1.50				−0.15	0.87	0.39				0.13	1.14	0.36
Nagelkerke Pseudo R²	0.05			0.10			0.04			0.07			0.04			0.17		
Chi² (df = 3 & df = 6)/ ^a^ Chi² (df = 2 & df = 5)	18.92***			33.75***			^a ^7.42*			^a ^12.62*			^a ^6.34*			^a^27.33** *		

* *p* < 0.05; ** *p* < 0.01; *** *p* < 0.001.

^1 ^no surgery specialty = 0; surgery specialty = 1; ^2 ^0 = male; 1 = female; ^3^ first stage = 0; second stage = 1; ^4^ 0 = not living together; 1 = living together.

^5 ^Controlled for gender x living together*: p > . .05*.

Gender differences in motives are found in Table6. It shows that gender contributes to profession-related motives and motives related to life-style for specialty preferences. Men value profession-related motives higher, whereas women value life-style motives higher than men.

**Table 6:** Multiple Regression analysis for gender, the stage of education and living together-related with profession-related motives and life-style motives for all students, first stage students and second stage students

	**All**				**First stage (N = 253)**				**Second stage (N = 209)**			
	**Profession- related motives (N = 1014)**		**Life-style motives (N = 1024)**		**Profession- related motives (N = 635)**		**Life-style motives (N = 644)**		**Profession- related motives (N = 379)**		**Life-style motives (N = 380)**	
	***β***	***t***	***β***	***t***	***β***	***t***	***β***	***t***	***β***	***t***	***β***	***t***
Gender ^1,4^	−0.19	−6.07*	0.18	5.91***	−0.21	4.83***	0.18	4.66***	−0.20	−3.95***	0.18	3.70***
Stage of education^2^	−0.05	−1.73	0.03	1.07								
Living together^3,4^	−0.01	−0.24	0.13	4.15***	−0.01	0.21	0.06	1.55	−0.01	−0.28	0.22	4.35***
R²	0.039		0.048		0.036		0.037		0.040		0.082	
∆ R²	0.036		0.045		0.033		0.034		0.035		0.078	

* *p* < 0.05; ** *p* < 0.01; *** *p* < 0.001.

^1 ^0 = male; 1 = female; ^2^ first stage = 0; second stage = 1; ^3^ 0 = not living together; 1 = living together.

^4 ^Controlled for gender x living together*: p > .05*.

### Stage of life, choice and motivation

The influence of the stage of education on the choice of continuing medical education (Model 1, Table4) and on medical specialty preference (Model 4, Table4) has not been found.

Living together with a partner influences the choice of continuing medical education (Model 1, Table4). Medical students living together with a partner are more likely to prefer general practice to a medical specialty, compared to students who are not living with a partner. This was also found for students in both stages of education.

Furthermore, medical students who are living with a partner are less likely to prefer a surgery specialty than a non-surgery specialty, compared to medical students who are not living with a partner (Model 3, Table5). This difference was not found for students in both stages separately.

The regression analysis (Table6) shows that whether or not living with a partner is not related to profession-bound motives, but it has a significant effect on life-style motivation. Medical students who are living together with a partner are more focused on life-style motives than those who are not living with a partner. Separate analyses showed that these results only hold for students in the second stage.

### Testing the model

Tables 4 and 5 show the results of binary logistic regression analysis. The models concerning all students have respectively 86.9%, 87.4%, 57.8% and 58.6% of the variables correctly allocated. The percentages of Model 3 and Model 4 can be characterized as relatively low, but they are still capable of being interpreted. The same characteristics were found for separate analyses for both stages of education. The Chi-square tests show that all models fit the data sufficiently. The proportion of explained variance (R ²) for Models 1 to 4 was successively 3%, 23%, 5% and 10% for the whole population. The proportions for the explained variance of separate analyses for students in both stages were in the same range.

*The choice of continuing medical education* (Model 1, Table4) is mainly explained by the variable living together with a partner, followed by the contribution of gender. Women living together with a partner are more likely to choose general practice. In separate analyses for both stages of education, the gender impact was not found. Only students living together with a partner are more likely to prefer general practice.

Regarding the mediating effects of motives, it is found that the choice of continuing medical education is largely influenced by life-style motives (Model 2, Table4). Life-style factors such as flexible working hours and having small children are related to the choice of general practice above medical specialties. Furthermore, intrinsic motives such as personal involvement are related to the preference for general practice, whereas profession-related motives such as status and high income are not related to the choice of general practice. The influence of life-style motives on the choice of general practice prevailed in analyses for both stages of education. But intrinsic motives that is personal involvement, was only related to the preference for general practice for first stage students. The second stage students’ choice of general practice had no relationship with profession-related motives.

For both gender and living with a partner, all three types of motives are mediating the relationship with the choice of continuing medical education. This is because after adding motives in Model 2 the effect of both gender and living with a partner disappeared. In separate analyses for the stage of education only for living with a partner, are the two types of motives mediating the choice of general practice. In both stages life-style motives were mediating that relationship, but, in addition, in the first stage, personal involvement, and in second stage, profession related, motives mediated the choice.

Although the effect of Model 3 is small it can be stated that in *the preference of medical specialty* (surgery or non-surgery), living with a partner, again, is the most influential factor followed by gender. Men without a partner prefer surgery specialties. For second stage students only gender influence was traced, saying that men prefer surgery specialties. For first stage students no influences for specialty preferences were found.

Considering the influence of motives profession-related motives and life-style motives determine the preferences. Profession-related motives such as status and high income and a low interest in life-style motives, such as flexible working hours, are both related to the preference for surgery specialties. These influences only hold for second stage students.

For gender both types of external motives, profession-related and life-style, are mediating the relationship with medical specialty preference and this was also found for second stage students. Men with a preference for surgery specialties are influenced by profession-related motives and not by life-style motives.

In the whole population the influence of living with a partner was not mediated by motives.

## Discussion

### Gender

The finding that men aspire more to surgery specialties and women prefer more often general practice and non-surgery specialties is consistent with previous research [3,6,8-14]. However, the significance of gender influence, stating the higher aspirations of women for general practice is not very strong (see Table4, Model 1: p = 0.046). In separate analyses of different stages of education this gender influence even disappeared.

Another finding was that no interaction effect was found between gender and living with a partner. Living with a partner seems to influence both male and female students in their preferences. These findings indicate that differences between male and female students in basic preferences are diminishing and family arguments are no longer only a female issue.

Students living with a partner may have other priorities, because they may be influenced by the ideas and expectations of their partner. This assumption is supported by the findings of Barshes et al. [29] showing that in case of a partner who does not support their career ambitions, married students prefer primary care above medical specialties. In addition, various studies show that married doctors often prefer general practice above a medical specialty, because of the higher compatibility with family life [1,3,4,8,29]. Future, research could focus in more detail on the role of the partner in the process of career choice.

### Motives

In types of motives men and women are differently oriented. Our results are consistent with findings stating that men are primarily motivated by income, status and technical aspects of work, while women are primarily motivated by life-style motives such as flexible working hours, and control over their life-style and domestic situation [9,25,27]. Surgery specialties are definitely dominated by men [9,30,34,35]. If living with a partner is as important for men as it is for women the coming generation of doctors will be more open-minded and support a cultural change in which surgery specialties are more accessible to women. Not all medical specialties facilitate the balance between home and work [1,36]. This situation may limit students with a partner in their choices, raising the possibility that they reconsider their initial choice and opt for a medical specialty more appropriate to their circumstances. Restricting medical students in their career choice because of the high demands in working conditions can be a negative strategy, which neglects talent and competencies [29,37].

Another issue is that motives are mediating the relationship between living with a partner and the choice of GP or medical specialty. Most students with a partner choose general practice, because it is more compatible with family life than a medical specialty [3,8,38]. For more clarity in the mediating effect of motives, longitudinal studies are necessary to find out to what extent motives change once someone has started to live together with a partner.

### Remarks and further research

Firstly, since medical specialties differ considerable in professional characteristics [39], in future research more detailed analyses of each individual specialty could show clear distinctions between various motives and their relationship to each specialty separately.

Secondly, this study is focused on basic medical education. Future studies should at least include students who are in specialist training, because the number of students living with a partner is rather small in this study [36,40].

Thirdly, this research has only focused on the needs and choices of respondents regarding their future careers. There are no data on the actual decisions they have taken or will take. For a better understanding of the choice process during medical education and employment, a cohort study on this subject is recommended. A fourth remark is about the factor analysis, which showed two different types of external motivation: professional and life-style oriented. This distinction was acceptable, because the content of the items were clearly focused on two domains (see addendum 1).

Finally, the present study provides a contribution to the existing knowledge of career aspirations of medical students. The research is innovative in that it focuses on the mediating effects of motivation on the relationship between gender and the stage of life on the one hand, and two types of choice in further medical career on the other hand. Moreover, this study highlights the differences between students who are living with a partner and those who are not. An interesting finding is the gender similarity in considering the family position as an important issue in medical preferences. The results establish a broader framework for the factors that are important in the selection process of medical students and therefore stimulate further research on this topic. In addition, given the fact that this investigation was aimed at medical students from all Dutch universities, the results can serve as a starting point for considerations in national policy.

## Conclusions

From the perspectives of gender, the stage of education and whether living, or not, with a partner, this study has provided insight into the influence on choices in medical education, especially the impact of several types of motivation. For all respondents, the results show that gender and living with a partner influence the choices for a continuation in GP or medical specialty training, next to the preferences in medical specialty. Moreover, external and intrinsic motives are mediating, to a greater or lesser degree, this relationship. In all analyses no interactions were found between gender and living with a partner, which means that considering living with a partner as an important influence in preferences is no longer specific female-related as it was in the past.

The results for first stage students show only influences on the choice of continuing medical education and no influences for preferences in medical specialty. For second stage students, the results show influences on both the choice of continuing medical education and specialty preferences.

## Addendum 1: Motivation items

Please indicate below how important each reason for your choice of specialty has been?

(5-point Likert-scale: very important – important – neutral – unimportant - very unimportant)

Interested in the field

Possibility of rotation in the field of subspecialty

Favorable circumstances at work

Learning special skills

High status

Career and promotion expectancies

High income

Domestic situation

Having small children

Control on life style

Patient oriented and meaningful

Availability of training

Flexible working hours

Achieving happiness

Contents of the future profession

## Competing interests

The authors declare that they have no competing interests.

## Authors' contributions

PH performed the statistical analyses, drafted the manuscript and contributed to all other aspects of the study.

## Pre-publication history

The pre-publication history for this paper can be accessed here:

http://www.biomedcentral.com/1472-6920/12/82/prepub
